# CRISPR/Cas9-Mediated Disruption of Duplicated Sizzled Genes Induces Twin-Tail-like Caudal Bifurcation in Goldfish (*Carassius auratus*)

**DOI:** 10.3390/ijms27167318

**Published:** 2026-08-16

**Authors:** Huijuan Li, Xiaoying Zhang, Xiaowen Wang, Rong Zhang, Lili Liu, Lixin Sun, Zhigang Yao, Hua Zhu

**Affiliations:** 1 Beijing Key Laboratory of Fishery Biotechnology, Fisheries Science Institute, Beijing Academy of Agriculture and Forestry Sciences, Beijing 100097, China; lihuijuan@baafs.net.cn (H.L.);; 2 Laboratory of Molecular Physiology, Department of Zoology, Kiel University, 24098 Kiel, Germany; 3 Fisheries College, Jimei University, Xiamen 361021, China

**Keywords:** goldfish, CRISPR/Cas9, sizzled, twin-tail phenotype

## Abstract

The twin-tail phenotype of goldfish represents a striking domestication-associated remodeling of the vertebrate caudal axial system and is classically linked to disruption of Chordin/BMP-mediated dorsal–ventral patterning. Although previous knockdown studies implicated sizzled (*szl*) in this process, genetic evidence from targeted disruption of endogenous *szl* loci remains limited. Here, we used CRISPR/Cas9 to mutate conserved coding regions shared by the duplicated goldfish paralogues *szlA* and *szlB* in single-tail embryos. Sanger sequencing and ICE analysis showed that *szl*-sgRNA2 and *szl*-sgRNA3 efficiently induced indels at both loci, whereas *szl*-sgRNA1 was ineffective. Across three independent biological replicates, twin-tail-like caudal bifurcation was observed in 44.63–48.19% of *szl*-sgRNA2-injected larvae, 69.47–79.61% of *szl*-sgRNA3-injected larvae, and 64.29–76.19% of larvae injected with the sgRNA mixture; *szl*-sgRNA1-injected larvae remained single-tailed. Calcein staining further revealed separation of distal caudal fin rays and partial splitting of the caudal skeletal complex in *szl*-edited larvae. qRT-PCR showed selective remodeling of dorsal–ventral patterning genes, including reduced *chdA* and eve1 expression and increased *bmp2* and *nog1* expression. These findings provide direct functional evidence that *szl* regulates median caudal patterning in goldfish and suggest that *szl*-dependent modulation of the Chordin/BMP network can generate twin-tail-like caudal morphology.

## 1. Introduction

Goldfish (*Carassius auratus*) represents one of the most remarkable examples of vertebrate morphological diversification under long-term artificial selection [1,2,3]. During domestication, numerous ornamental strains with highly divergent body shapes, fin morphologies, pigmentation patterns, and cranial structures have been established, making goldfish an important model for understanding how artificial selection reshapes developmental programs [1,3,4]. Among the numerous ornamental traits established during domestication, the twin-tail phenotype, which was first documented in 1596 CE after approximately 600 years of goldfish domestication for ornamental purposes, is particularly exceptional because it involves a lateral bifurcation of the caudal fin together with duplication and reorganization of the caudal axial skeletal system [4,5,6]. In most vertebrates, axial skeletal elements are arranged along the body midline, and the teleost caudal fin skeleton is normally positioned in the sagittal plane; in contrast, twin-tail goldfish possess bilaterally duplicated caudal fin rays and associated axial skeletal elements, representing a rare case in which the highly conserved midline organization of the vertebrate axial skeleton has been dramatically modified and stably maintained under domestication [6,7]. The rarity of laterally bifurcated caudal axial skeletons in nature further implies that twin-tail formation was caused by an uncommon developmental genetic change capable of producing large-scale morphological reorganization while retaining sufficient viability for domesticated propagation [6,7]. The rarity of laterally bifurcated caudal axial skeletons in nature further implies that twin-tail formation was caused by an uncommon developmental genetic change capable of producing large-scale morphological reorganization while retaining sufficient viability for domesticated propagation [6,7].

Molecular genetic studies have established that the classical twin-tail phenotype in goldfish is fundamentally associated with perturbation of embryonic dorsal–ventral patterning [6,7]. Abe et al. identified two duplicated *chordin* paralogues, *chdA* and *chdB*, and demonstrated that a twin-tail-specific nonsense mutation in *chdA* (*chdA*^E127X^) introduces a premature stop codon at amino acid position 127, producing a truncated Chordin protein lacking three of the four conserved cysteine-rich domains required for BMP antagonism and dorsal organizer activity [6,8]. Mechanistically, loss of functional *chdA* weakens Chordin-mediated BMP antagonism, leading to ventral tissue expansion, bilateral displacement of caudal fin-fold primordia, and subsequent formation of bifurcated fin folds and duplicated caudal axial structures [4,6]. However, unlike severe *chordin*-deficient mutants in other vertebrates, twin-tail goldfish retain relatively high viability, probably because the duplicated *chdB* paralogue partially compensates for dorsal organizer function and prevents excessive anterior–dorsal defects [7,9,10,11]. Thus, the *chdA*^E127X^ mutation represents a rare developmental genetic change that simultaneously produces large-scale caudal axial reorganization and maintains sufficient viability, allowing this allele to be positively selected and genetically fixed in ornamental twin-tail goldfish populations [6,7].

Although *chdA*^E127X^ is regarded as the major genetic route underlying the established ornamental twin-tail phenotype, dorsal–ventral patterning is regulated by a broader gene network, and *sizzled* (*szl*) has emerged as an important candidate gene for an alternative twin-tail-forming mechanism [12,13]. In zebrafish, mutation of *ogon/sizzled* can induce twin-tail-like morphology and exhibits higher viability than *dino/chordin* mutants, suggesting that *szl* disruption may alter caudal patterning without causing the severe developmental lethality often associated with complete *chordin* deficiency [9,14,15]. In goldfish, Abe et al. identified at least two duplicated *szl* paralogues, *szlA* and *szlB*, which likely originated from the lineage-specific genome duplication shared by goldfish and common carp [13]. These paralogues show differentiated expression patterns during early embryogenesis, with *szlA* displaying broader or stronger expression in the ventral region than *szlB* [13]. Morpholino-mediated depletion of *szl* in single-tail goldfish reproduced bifurcated caudal fin folds and bifurcated caudal fin rays resembling those of twin-tail goldfish [13]. Notably, *szlA* depletion produced stronger and more diverse dorsal–ventral patterning defects, whereas *szlB* depletion showed lower expressivity, indicating functional divergence between the two paralogues [13]. Furthermore, *szlA* depletion altered the expression of dorsal–ventral patterning genes, including reduced *chdA/chdB* expression and changes in *bmp2* expression, supporting the hypothesis that *szl* participates in the regulatory network controlling caudal fin-fold bifurcation [13,16,17].

Despite these findings, the functional role of *szl* in goldfish twin-tail formation remains to be further validated at the genomic level. Previous studies mainly relied on transient knockdown approaches, leaving unresolved whether targeted disruption of endogenous *szl* loci is sufficient to induce twin-tail-like caudal morphology and alter the associated dorsal–ventral patterning network [13]. In the present study, we used CRISPR/Cas9-mediated mutagenesis to disrupt *szl* in single-tail goldfish and systematically examined the resulting caudal fin phenotypes and expression patterns of dorsal–ventral axis-related genes. By integrating targeted genome editing, phenotypic assessment, and gene-expression analysis, this study provides direct functional evidence for the involvement of *szl* in goldfish caudal patterning and further clarifies its potential role as an alternative genetic route contributing to twin-tail formation beyond the classical *chdA*-dependent pathway.

## 2. Results and Discussion

### 2.1. Validation of CRISPR/Cas9-Mediated Editing at the Duplicated szl Loci

**Figure 1:**
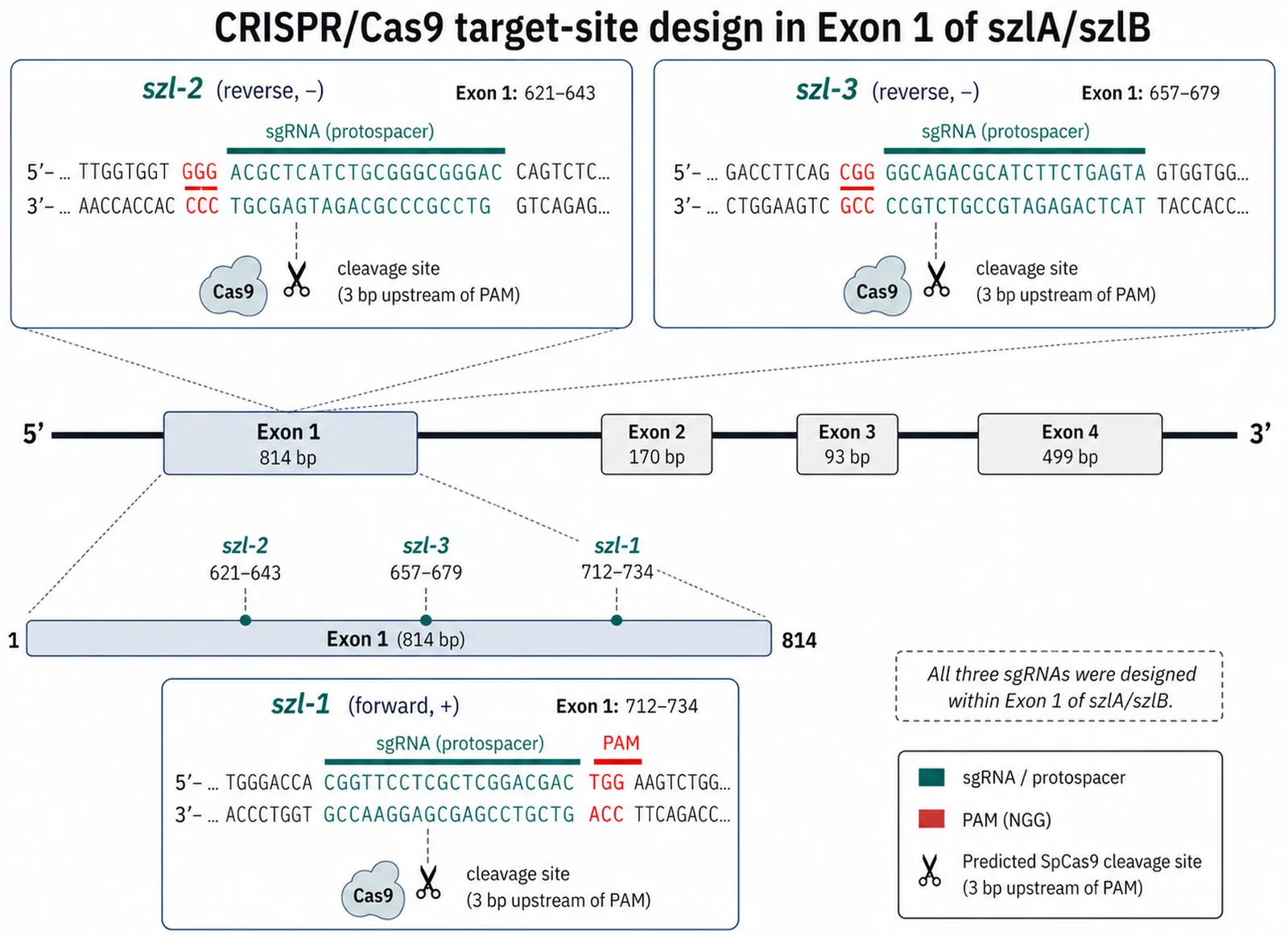
CRISPR/Cas9 target-site design within exon 1 of the duplicated goldfish *szlA*/*szlB* genes. Schematic representation of the exon organization of the goldfish *szlA*/*szlB* coding region and the positions of three sgRNA target sites designed for CRISPR/Cas9-mediated mutagenesis. All three sgRNAs were located within exon 1. The target sites *szl*-2 and *szl*-3 were designed on the reverse strand and located at positions 621–643 and 657–679 of exon 1, respectively, whereas *szl*-1 was designed on the forward strand and located at positions 712–734. Enlarged sequence diagrams show the sgRNA protospacer sequences, adjacent NGG PAM motifs, strand orientation and predicted SpCas9 cleavage sites, which occur approximately 3 bp upstream of the PAM sequence. Green bars indicate sgRNA protospacer sequences, red bases indicate PAM motifs, and scissors indicate predicted Cas9 cleavage positions.

**Figure 2:**
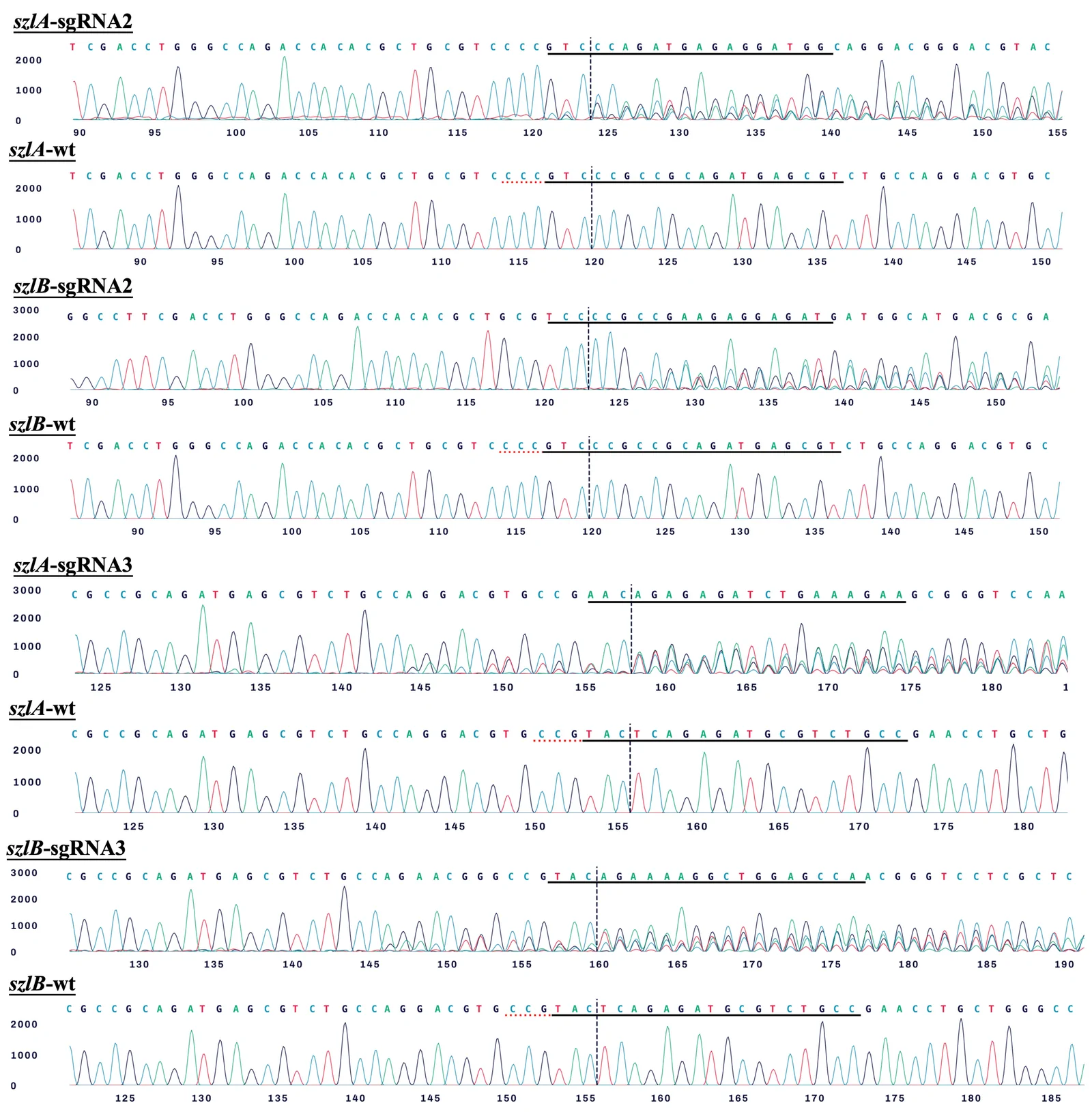
Sanger sequencing validation of CRISPR/Cas9-mediated mutagenesis at the duplicated goldfish *szlA* and *szlB* loci. Representative Sanger sequencing chromatograms showing targeted mutations induced by *szl*-sgRNA2 and *szl*-sgRNA3 at the corresponding ***szlA*** and ***szlB*** loci. Wild-type chromatograms are shown as controls for each target region. In the injected groups, obvious overlapping peaks appear downstream of the predicted Cas9 cleavage sites, indicating the presence of CRISPR/Cas9-induced indel mutations generated by non-homologous end joining repair. Black horizontal lines indicate the sgRNA protospacer sequences, red dashed lines indicate the PAM motifs, and vertical dashed lines mark the predicted Cas9 cleavage positions. Compared with the clean single peaks observed in wild-type controls, the mixed sequencing signals in *szlA*-sgRNA2, *szlB*-sgRNA2, *szlA*-sgRNA3, and szlB-sgRNA3 confirm efficient genome editing at both duplicated *szl* paralogues.

To evaluate the CRISPR/Cas9 editing efficiency of three sgRNAs designed against the conserved coding region shared by the paralogous genes *szlA* and *szlB* (Figure 1), Sanger sequencing was performed on PCR-amplified target loci from injected embryos. Among the three designed guides, both *szl*-sgRNA2 and *szl*-sgRNA3 efficiently induced targeted genome editing in injected embryos, as evidenced by clear overlapping chromatogram peaks at the expected Cas9 cleavage sites adjacent to the PAM sequence (Figure 2). Quantitative analysis showed that 75% (22/30) of samples in the *szl*-sgRNA2 group and 80% (24/30) in the *szl*-sgRNA3 group carried detectable indel mutations, consistent with high-efficiency non-homologous end joining (NHEJ)-mediated repair at the target locus. In contrast, *szl*-sgRNA1 failed to induce detectable genome editing, as all sequenced samples exhibited wild-type chromatogram profiles without overlapping peaks or indel-associated signal disruption at the target site. Collectively, these results demonstrate that sgRNA-2 and sgRNA-3 exhibit robust and reproducible editing efficiency at the *szl* target locus, whereas sgRNA-1 is ineffective under the present experimental conditions.

The marked differences among the three sgRNAs indicate that editing performance was strongly target dependent. The absence of detectable mutations in sgRNA1-injected embryos, together with the robust activities of sgRNA2 and sgRNA3, is consistent with the known influence of local sequence context and target accessibility on CRISPR/Cas9 efficiency [18]. Importantly, sgRNA1 also failed to produce caudal bifurcation, whereas the effective guides generated the phenotype described below, supporting a specific association between *szl* mutagenesis and the observed developmental defects rather than nonspecific effects of microinjection.

### 2.2. Characterization of Indel Mutations Induced by szl-sgRNAs

**Figure 3:**
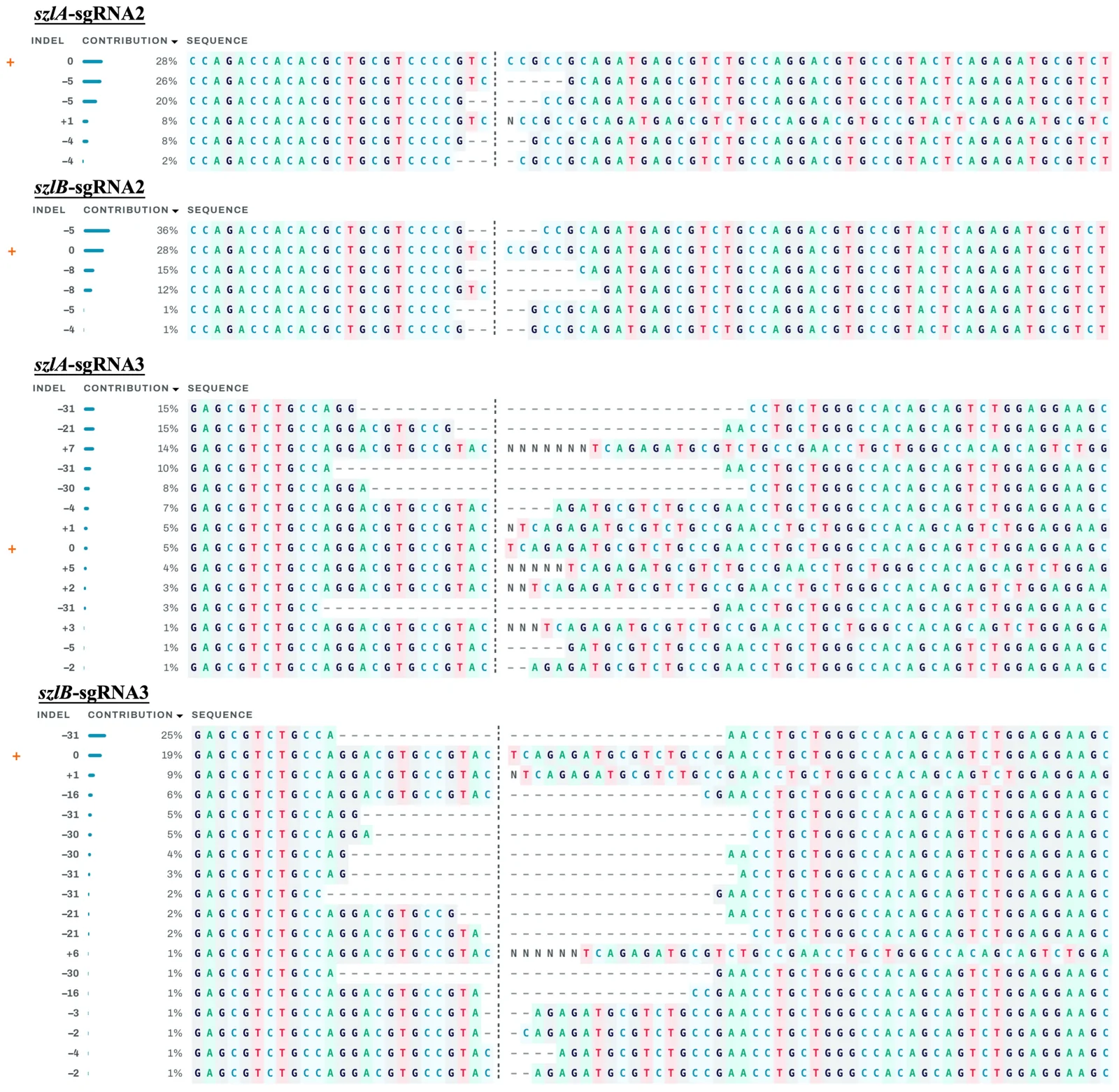
ICE-based characterization of indel mutation profiles induced by *szl*-sgRNA2 and *szl*-sgRNA3 at the duplicated goldfish *szlA* and *szlB* loci. Representative ICE decomposition results showing the spectrum and relative contribution of CRISPR/Cas9-induced indel mutations at the *szlA*-sgRNA2, *szlB*-sgRNA2, *szlA*-sgRNA3, and *szlB*-sgRNA3 target sites. For each target locus, the left column indicates the predicted indel size, the middle column shows the estimated contribution of each allele class, and the right panel displays the inferred sequence alignment around the Cas9 cleavage site. Deleted nucleotides are indicated by dashed lines, inserted or ambiguous bases are shown within the aligned sequences, and the vertical dashed line marks the predicted cleavage position.

The mutation profiles induced by *szl*-sgRNA2 and *szl*-sgRNA3 at the target sites of *szlA* and *szlB* were further characterized using ICE analysis (Figure 3 and Figure S1). Both sgRNAs generated multiple types of indel mutations at the predicted Cas9 cleavage sites, confirming efficient CRISPR/Cas9-mediated mutagenesis in the shared target regions of the two paralogous *szl* genes. The ICE decomposition profiles showed clear divergence between the edited and control chromatograms within the inference windows, with high model fitting values for *szlA*-sgRNA2 (R^2^ = 0.92), *szlB*-sgRNA2 (R^2^ = 0.93), *szlA*-sgRNA3 (R^2^ = 0.92), and *szlB*-sgRNA3 (R^2^ = 0.89), supporting the reliability of the inferred indel spectra (Figure S1).

For *szlA*-sgRNA2, two distinct −5 bp deletion classes accounted for 26% and 20% of the inferred alleles, respectively, representing 46% in total. Two distinct −4 bp deletion classes contributed 8% and 2%, respectively, representing 10% in total, while a +1 bp insertion accounted for 8%. Unedited alleles represented approximately 28% of the analyzed sequences. For *szlB*-sgRNA2, the mutation spectrum was similarly dominated by short deletions. A −5 bp deletion accounted for approximately 37%, whereas two distinct −8 bp deletion classes accounted for approximately 15% and 12%, respectively, representing 27% in total. Unedited alleles represented approximately 28%, and the remaining minor deletion classes occurred at low frequencies (Figure 3). These results indicate that *szl*-sgRNA2 mainly induced short deletion mutations at both *szlA* and *szlB* loci, with insertion events occurring only rarely.

Compared with *szl*-sgRNA2, *szl*-sgRNA3 produced a broader and more heterogeneous indel spectrum, characterized by larger deletions and multiple minor insertion/deletion classes. At the *szlA*-sgRNA3 target site, the most abundant mutation class was a −31 bp deletion, accounting for approximately 28% of the indel mixture, followed by a −21 bp deletion at approximately 15% and a −30 bp deletion at approximately 8%. Additional deletion events, including −5 bp, −4 bp, −2 bp, and −1 bp deletions, were detected at lower frequencies. Several insertion events were also observed, including +7 bp, +5 bp, +3 bp, +2 bp, and +1 bp insertions, with the +7 bp insertion representing the most prominent insertion class at approximately 14%. Unedited alleles accounted for only approximately 5% of the analyzed sequences. At the *szlB*-sgRNA3 target site, the mutation profile was also dominated by large deletions. The −31 bp deletion was the most abundant mutation type, accounting for approximately 35% of the alleles, followed by a −30 bp deletion accounting for approximately 10%. Additional deletion events included −21 bp, −16 bp, −4 bp, −3 bp, and −2 bp deletions, each contributing at lower frequencies. Insertion events were less frequent, with a +1 bp insertion accounting for approximately 9% and a +6 bp insertion detected at approximately 1%. Unedited alleles accounted for approximately 19% of the analyzed sequences. The indel size distribution further confirmed that *szl*-sgRNA3 preferentially induced larger deletions, especially −31 bp, −30 bp, and −21 bp events, whereas *szl*-sgRNA2 mainly generated short deletions around the cleavage site (Figure 3).

Collectively, these ICE-based analyses demonstrate that *szl*-sgRNA2 and *szl*-sgRNA3 both efficiently induced indel mutations at the *szlA* and *szlB* target loci, but produced distinct mutation profiles. *szl*-sgRNA2 predominantly generated short deletion-type mutations, whereas *szl*-sgRNA3 induced larger and more complex indel events, particularly long deletions. These findings highlight sgRNA-dependent differences in DNA repair outcomes and further support the use of *szl*-sgRNA2 and *szl*-sgRNA3 for functional disruption of the duplicated *szlA*/*szlB* genes in goldfish.

The distinct mutation spectra produced by sgRNA2 and sgRNA3 are consistent with target-dependent repair outcomes following Cas9 cleavage [18,19]. sgRNA2 predominantly produced short deletions, whereas sgRNA3 generated a broader distribution that included recurrent large deletions. Because many of these alterations are predicted to disrupt the coding sequence, both guides are expected to reduce *szl* function. The larger and more heterogeneous lesions induced by sgRNA3 may contribute to its stronger phenotypic effect; however, this interpretation remains provisional because genotype-phenotype relationships were not determined in individual larvae and the F0 animals were likely mosaic.

### 2.3. szl Disruption Induces Bifurcated Caudal Fin-Fold Morphology in Goldfish Larvae

**Figure 4:**
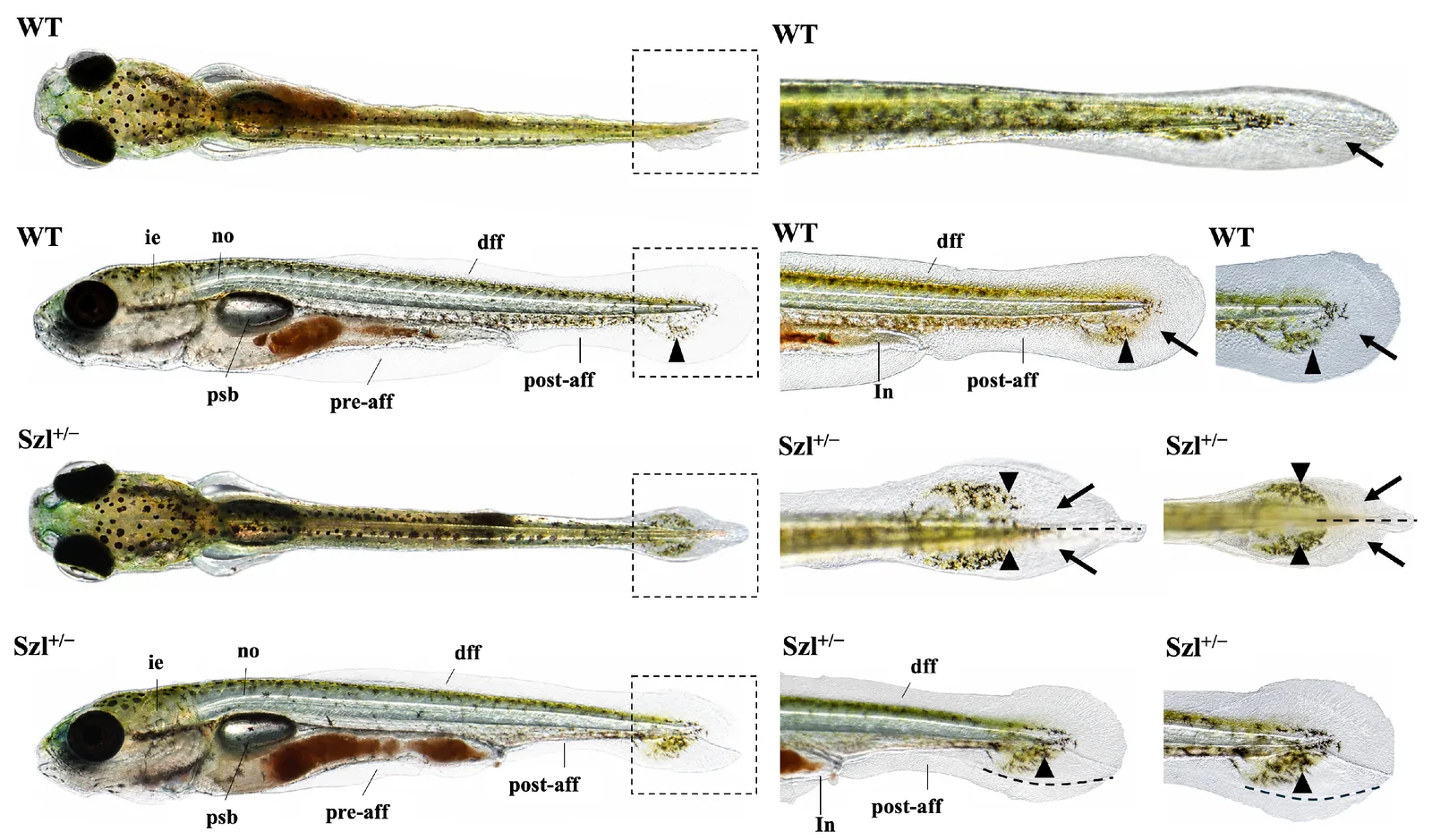
Loss of *szl* function induces bifurcation of the caudal fin fold in goldfish larvae at the posterior swim bladder stage. Representative bright-field images showing caudal fin-fold morphology in wild-type (WT) and *szl*-edited goldfish larvae at the posterior swim bladder stage. WT larvae exhibited a typical single-tail morphology, with a straight notochord and a continuous median caudal fin fold extending along the sagittal plane. Enlarged views of the posterior trunk and caudal region show an intact dorsal fin fold and postanal fin fold converging into a single distal caudal fin-fold domain. In contrast, *szl*-edited larvae displayed an expanded and bifurcated distal caudal fin fold, characterized by bilateral separation of caudal fin-fold tissue and deviation of the posterior tail axis. Black triangles indicate the caudal fin-fold domains adjacent to the posterior axial region; in *szl*-edited larvae, paired black triangles highlight the bilaterally separated caudal fin-fold domains. Arrows indicate the bifurcated distal caudal fin-fold region, and dashed lines outline the altered posterior axis or fin-fold boundary. Boxed regions in whole-larva images indicate the areas magnified in the corresponding panels. Abbreviations: ie, inner ear; dff, dorsal fin fold; post-aff, postanal fin fold; pre-aff, preanal fin fold; psb, posterior swim bladder; no, notochord.

**Figure 5:**
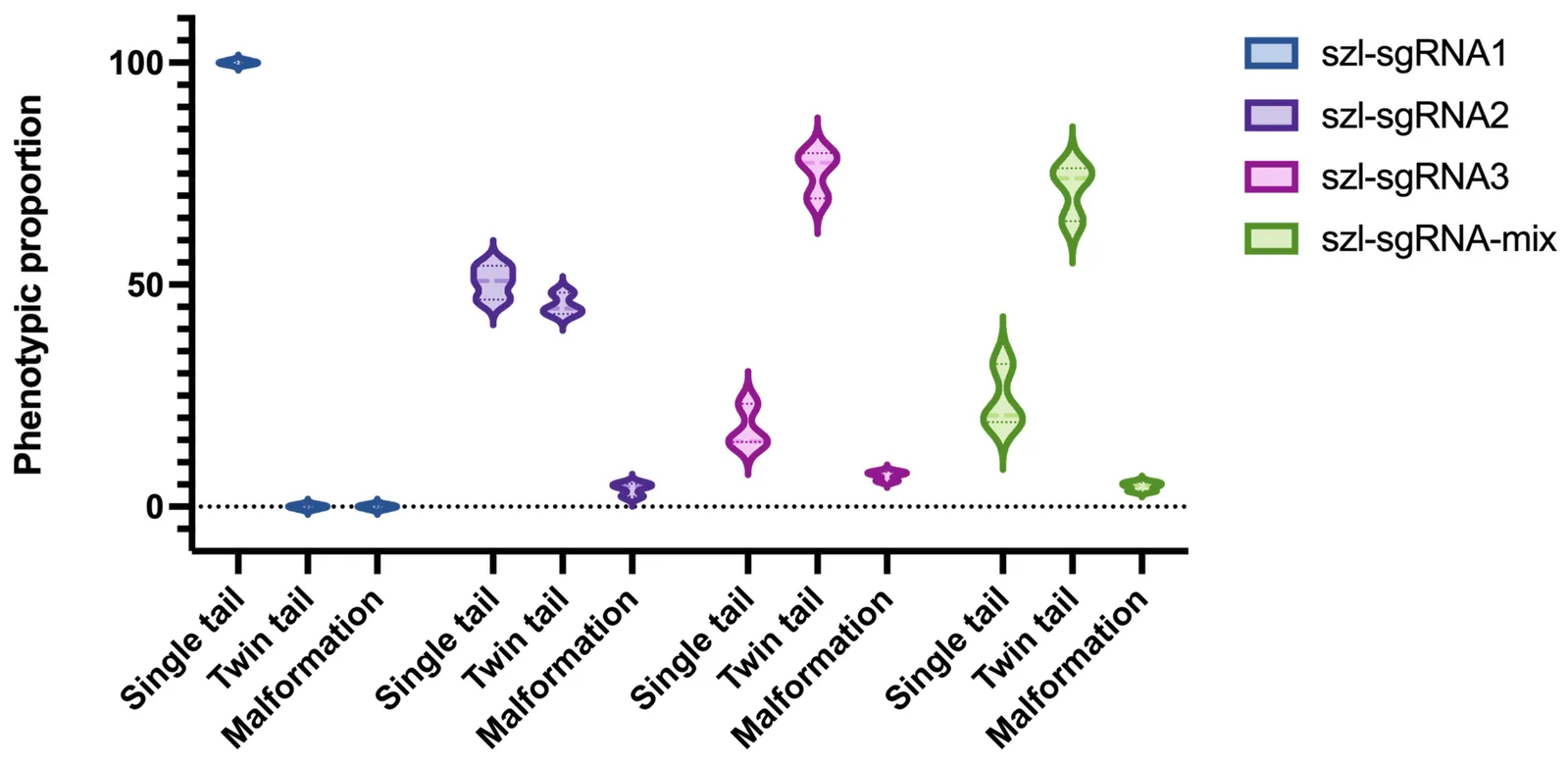
Quantitative analysis of caudal phenotypic outcomes following CRISPR/Cas9-mediated disruption of *szl* in goldfish larvae. Violin plots showing the proportions of single-tail, twin-tail-like and malformed larvae in embryos injected with *szl*-sgRNA1, *szl*-sgRNA2, *szl*-sgRNA3 or the combined *szl*-sgRNA-mix. Each dot represents one biological replicate, and dashed lines within each violin indicate the distribution and central tendency of the phenotypic proportions. Injection with *szl*-sgRNA1, which showed no detectable editing activity, produced only single-tail larvae. In contrast, *szl*-sgRNA2, *szl*-sgRNA3 and *szl*-sgRNA-mix induced clear twin-tail-like caudal bifurcation, with *szl*-sgRNA3 and the sgRNA-mix group producing higher proportions of bifurcated larvae than *szl*-sgRNA2.

At the posterior swim bladder stage (Psb stage), wild-type goldfish larvae exhibited a typical single-tail morphology, characterized by an inflated posterior swim bladder, a straight notochord and continuous dorsal, preanal and postanal fin folds (Figure 4). The posterior notochord extended along the midline into the caudal region, and the distal fin-fold tissue formed a single median caudal fin fold at the end of the caudal peduncle. These features are consistent with the established morphological criteria for Psb-stage goldfish larvae, during which the posterior swim bladder is visible, the notochord remains straight and median fin folds are still retained. In contrast, *szl*^+^/^−^ edited larvae showed an evident alteration in caudal fin-fold organization at the same stage. The distal caudal fin fold was expanded and separated into two lobular domains, forming a forked or bifurcated caudal fin-fold phenotype. Magnified views further revealed displacement of the caudal fin-fold tissue and apparent broadening or deviation of the posterior tail axis, suggesting the early establishment of a bifurcated caudal pattern. Phenotypic quantification showed that *szl*-sgRNA1 did not induce detectable tail bifurcation, with 100% of larvae classified as single-tail in all three replicates. Across three independent biological replicates, twin-tail-like caudal bifurcation was observed in 44.63–48.19% of *szl*-sgRNA2-injected larvae, 69.47–79.61% of *szl*-sgRNA3-injected larvae, and 64.29–76.19% of larvae injected with the sgRNA mixture, whereas *szl*-sgRNA1-injected larvae remained single-tailed (Figure 5). Malformed larvae were defined as individuals exhibiting nonspecific developmental abnormalities other than caudal fin bifurcation. No reproducible mild dorsalization or obvious anterior-dorsal abnormalities were observed; the predominant phenotype was restricted to the posterior caudal region.

Sanger sequencing showed clear sgRNA-dependent editing activity. *szl*-sgRNA2 and *szl*-sgRNA3 efficiently induced mutations at the conserved target regions of *szlA* and *szlB*, whereas *szl*-sgRNA1 showed no detectable editing. This molecular difference was consistent with the phenotypic outcome: sgRNA1-injected larvae remained entirely single-tailed, while sgRNA2, sgRNA3 and the sgRNA-mix groups produced bifurcated caudal fin-fold phenotypes. In particular, sgRNA3 induced a higher frequency of double-tail larvae than sgRNA2, and the sgRNA-mix group maintained a similarly high bifurcation rate. The close correspondence between editing efficiency and phenotype strongly supports a causal relationship between *szl* disruption and caudal bifurcation, rather than nonspecific injection damage. ICE analysis further showed that sgRNA2 and sgRNA3 generated different indel profiles. sgRNA2 predominantly induced short deletions, whereas sgRNA3 produced larger and more heterogeneous indels, including frequent −31 bp, −30 bp and −21 bp deletions. Since Cas9-induced double-strand breaks are repaired mainly through error-prone end-joining pathways, such target-dependent indel outcomes are expected [18,19]. Functionally, larger deletions may have stronger disruptive effects on coding sequence integrity than small indels. This may explain why sgRNA3, which generated more complex deletion profiles, produced a higher proportion of bifurcated larvae. The higher frequency of caudal bifurcation observed in the sgRNA3 group is more likely associated with its greater overall editing efficiency and the higher proportion of mutant sequences generated at both *szlA* and *szlB* loci. In duplicated genes such as *szlA* and *szlB*, the final phenotype is likely determined by the combined mutational burden across both paralogues.

### 2.4. szl Disruption Causes Persistent Bifurcation of Caudal Skeletal Patterning

**Figure 6:**
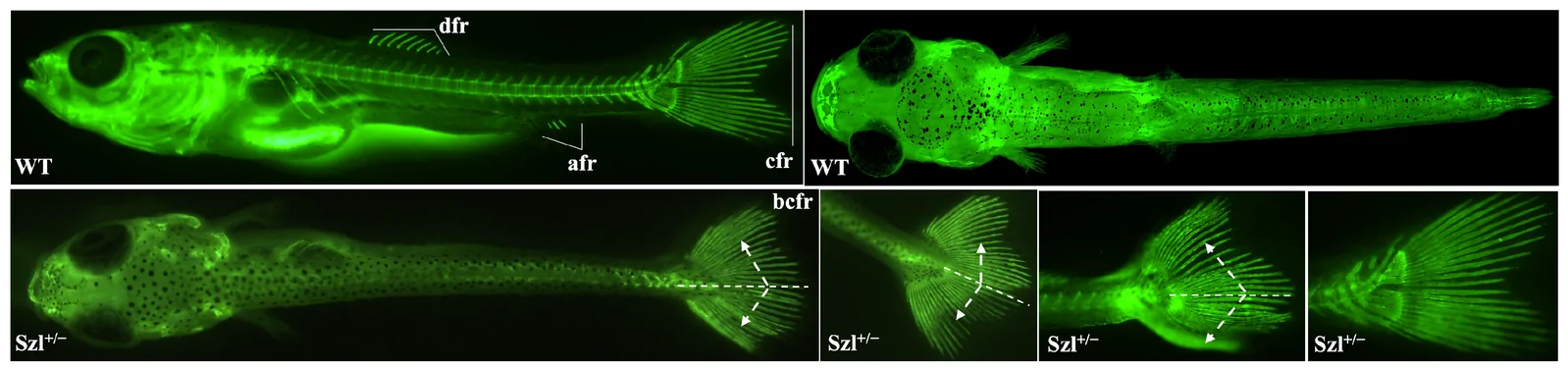
Calcein staining reveals bifurcated caudal fin ray patterning in *szl*-edited goldfish larvae. Representative whole-body and enlarged caudal images of calcein-stained wild-type (WT) and *szl*-edited larvae at the anal fin ray stage. WT larvae exhibited a continuous axial skeleton, regularly arranged dorsal and anal fin rays, and a single median caudal fin ray complex. In contrast, *szl*-edited larvae displayed separation of the distal caudal fin rays into two divergent domains, forming a bifurcated caudal fin ray pattern. White arrows indicate the separated caudal fin ray branches, and white dashed lines outline their divergent arrangement. Abbreviations: afr, anal fin ray; bcfr, bifurcated caudal fin ray; cfr, caudal fin ray; dfr, dorsal fin ray.

To further evaluate the effect of *szl* disruption on caudal fin skeletal development, calcein green staining was performed in goldfish larvae at the anal fin ray stage (Afr stage) (Figure 6). At this stage, WT larvae exhibited a continuous axial skeleton, with regularly arranged neural spines, dorsal fin rays, anal fin rays and caudal fin rays along the sagittal body plane. The caudal fin rays formed a single, symmetrical fan-shaped structure, and the hypural elements, parhypural and lepidotrichia, were compactly organized in the distal caudal skeleton. These features are consistent with normal goldfish skeletal development at the Asb stage, when dorsal and anal fin condensation is enhanced and neural spines become relatively highly calcified. In contrast, *szl*^+/−^ larvae displayed evident disruption of caudal skeletal organization. The distal caudal fin rays were separated into two divergent domains rather than forming a single median fan, accompanied by deviation of the posterior tail axis and partial splitting of the caudal skeletal complex. This bifurcated caudal fin ray pattern resembles the twin-tail goldfish phenotype, in which duplicated caudal fin structures and laterally bifurcated axial skeletal elements are formed. Together with the Afr-stage morphological observations, these results indicate that *szl* disruption causes persistent defects in posterior fin-fold and caudal skeletal patterning, promoting the formation of a double-tail-like caudal phenotype during goldfish larval development.

*Szl*-edited larvae displayed expansion and separation of the distal caudal fin fold into two lobular domains, whereas wild-type larvae retained a single median caudal fin fold. This phenotype closely resembles the early developmental morphology of twin-tail goldfish. The classical twin-tail phenotype is caused by a loss-of-function mutation in one duplicated chordin paralogue, *chdA/chdS*, which alters embryonic dorsal–ventral patterning and induces bifurcation of the embryonic fin fold and caudal axial skeleton [6]. Developmental studies further showed that early fin-fold bifurcation is a precursor of duplicated caudal fins and bifurcated axial skeletal elements in twin-tail goldfish [3,4]. Therefore, *szl* disruption likely affects the same posterior median fin-forming field involved in twin-tail development. Calcein skeletal staining confirmed that early fin-fold bifurcation was followed by abnormal caudal skeletal organization. In wild-type larvae, caudal fin rays formed a single fan-shaped structure along the sagittal plane, whereas *szl*-edited larvae showed separation of distal caudal fin rays and partial splitting of the caudal skeletal complex. This progression indicates that *szl* disruption affects an upstream patterning event that is later translated into skeletal architecture. These findings are consistent with goldfish larval staging studies and developmental analyses of twin-tail goldfish, which show that changes in early fin-fold organization can lead to bifurcated median fin and caudal skeletal structures [3,20].

### 2.5. szl Disruption Selectively Alters Dorsal–Ventral Patterning-Related Gene Expression

**Figure 7:**
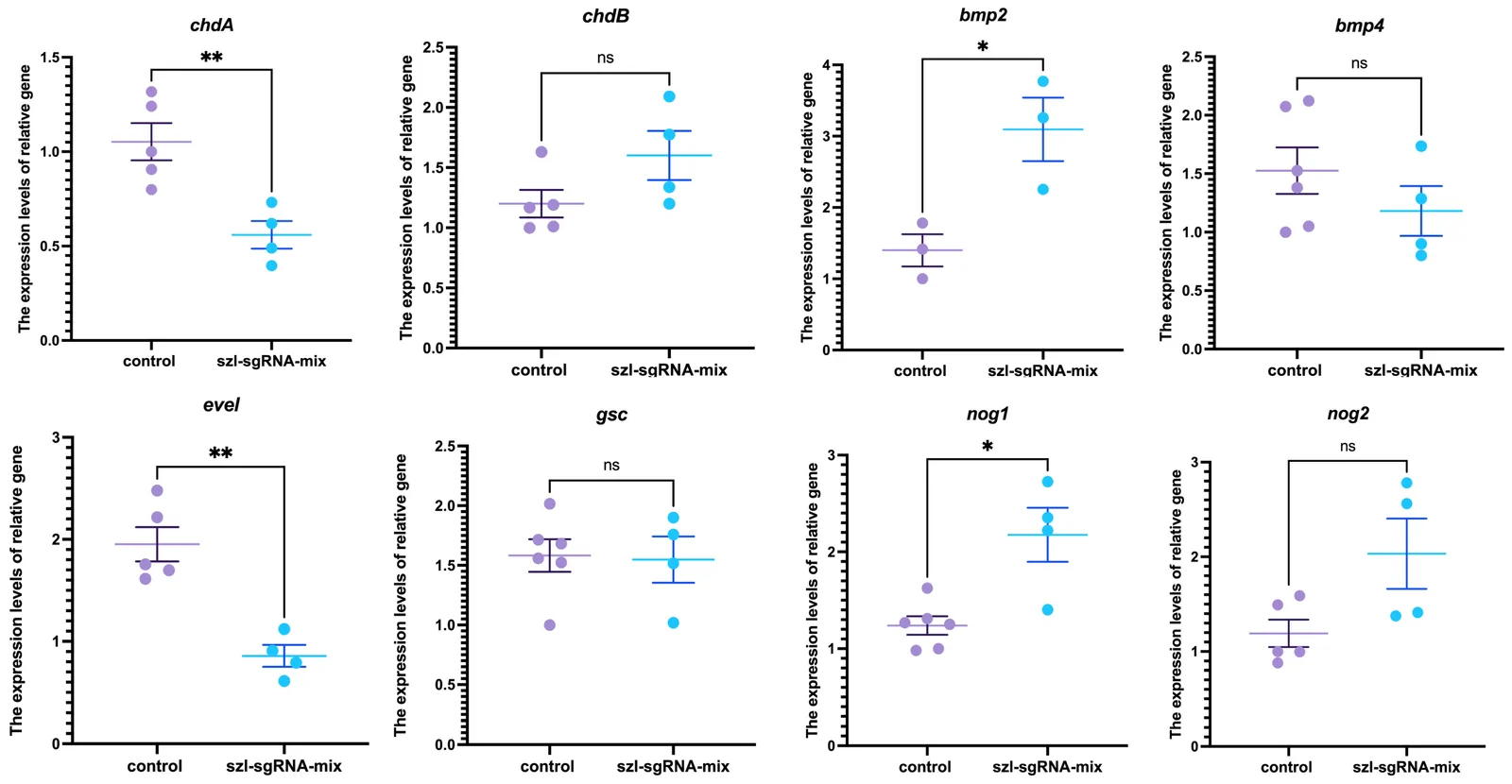
Expression changes in dorsal–ventral patterning-related genes in *szlA*/*szlB* co-edited goldfish larvae. Quantitative RT-PCR analysis of *chdA*, *chdB*, *bmp2*, *bmp4*, *eve1*, *gsc*, *nog1*, and *nog2* in control and edited caudal tissues. Relative expression levels were normalized to *β-actin* and calculated using the 2^−ΔΔCt^ method. Each edited biological replicate consisted of a pooled sample from three phenotypically selected larvae carrying detectable mutations at both *szlA* and *szlB*; three to four independently prepared edited pools were analyzed together with independently prepared control samples. Each dot represents one biological replicate, and horizontal bars indicate the mean ± SEM. Statistical significance was determined using a two-tailed Student’s *t*-test; * *p* < 0.05, ** *p* < 0.01; ns, not significant.

To further examine whether the caudal fin abnormalities observed in *szl*-edited larvae were accompanied by changes in dorsal–ventral patterning-related gene expression, qRT-PCR was performed to analyze representative genes associated with the Chordin/BMP signaling network (Figure 7). Compared with the control group, *szl*-sgRNA-mix larvae showed a significant reduction in the expression of *chdA*, whereas the expression of its paralogue *chdB* was not significantly altered. Among BMP-related genes, *bmp2* was significantly upregulated in the *szl*-sgRNA-mix group, while *bmp4* showed no significant difference between the two groups. In addition, the expression of *eve1* was significantly decreased after *szl* disruption, whereas *gsc* remained unchanged. For BMP antagonists, *nog1* was significantly increased in *szl*-edited larvae, while *nog2* showed an upward trend but did not reach statistical significance. Together, these qRT-PCR results show that *szl* disruption is associated with selective changes in dorsal–ventral patterning-related gene expression, particularly involving *chdA*, *bmp2*, *eve1* and *nog1*, while several other genes in this regulatory network, including *chdB*, *bmp2*, *gsc* and *nog2*, were not significantly affected at the examined stage.

Mechanistically, the *szl* phenotype is best explained by disruption of the extracellular Chordin/BMP regulatory network. In vertebrate embryos, BMP signaling promotes ventral cell fate, while Chordin antagonizes BMP activity to establish dorsal–ventral polarity [9,10,21]. Sizzled/Ogon, the zebrafish orthologue of *szl*, functions as a BMP-feedback regulator and stabilizes Chordin by inhibiting Tolloid-family metalloproteinases that cleave Chordin [17,22]. Thus, loss of *szl* would be expected to weaken Chordin-dependent BMP antagonism. No reproducible mild dorsalization or obvious anterior-dorsal abnormalities were observed in the *szl*-edited larvae, and the predominant phenotype was restricted to the posterior caudal region. Although *szl* and *chdA* both participate in the Chordin/BMP regulatory network, they act at different regulatory levels. Chordin directly binds to and antagonizes BMP ligands, whereas Sizzled indirectly maintains Chordin activity by inhibiting BMP1/Tolloid-mediated Chordin cleavage. Therefore, *chdA* deficiency directly reduces BMP antagonism, whereas *szl* disruption primarily affects Chordin stability and availability. This mechanistic distinction may explain why *szl*-edited larvae developed a twin-tail-like caudal phenotype without the broader dorsal–ventral abnormalities associated with *chdA* deficiency. The qRT-PCR results support this model. *chdA* was significantly downregulated after *szl* disruption, whereas *chdB* was not significantly changed. This paralogue-specific response is notable because *chdA/chdS* is the causal gene underlying the classical twin-tail phenotype, while *chdB* may retain compensatory developmental activity [6,23,24]. The absence of a significant change in *chdB* transcript abundance in the present study differs from the early whole-embryo morpholino observations by Abe et al. [13]. This difference may reflect developmental stage, tissue source, disruption strategy, and analytical method: the present analysis quantified bulk transcript abundance in dissected caudal tissue from later-stage CRISPR/Cas9-edited larvae, whereas the earlier study assessed spatial expression after morpholino-mediated *szlA* knockdown. Moreover, because Sizzled primarily regulates Chordin protein stability, unchanged *chdB* mRNA abundance does not exclude altered Chordin activity or BMP signaling. F0 mosaicism also limits definitive genotype-specific interpretation. Stable *szl* mutant families will therefore be required to verify heritability, establish genotype-phenotype relationships, and distinguish the respective functions of *szlA* and *szlB*; such lines may also provide useful germplasm resources for double-tail goldfish breeding. Increased *bmp2* expression is consistent with altered BMP signaling, while increased *nog1* may represent a compensatory response because Noggin proteins also antagonize BMP activity [25]. The decrease in *eve1* further indicates that the transcriptional response was selective rather than a uniform activation of ventral markers and may reflect stage-specific secondary or compensatory regulation.

## 3. Materials and Methods

### 3.1. Experimental Animals and Ethics Statement

Single-tail goldfish broodstock were maintained under standard aquaculture conditions. Fertilized embryos were collected immediately after natural spawning and staged according to established goldfish developmental criteria. All procedures involving goldfish were reviewed and approved by the Ethics Committee of the Beijing Academy of Agriculture and Forestry Sciences [approval number BAAFS-KJLL-20250909 and approval date 9 September 2025] and were performed in accordance with the institutional guidelines for the care and use of laboratory animals.

### 3.2. Design and Synthesis of sgRNAs

Within the goldfish genome, two *sizzled* paralogues, *szlA* and *szlB*, were identified. To simultaneously disrupt *szlA* and *szlB*, sgRNAs were designed to target the conserved regions shared by these two paralogues. Three sgRNAs were designed within the common sequence of *szlA* and *szlB* using the online CRISPOR tool (http://crispor.tefor.net/, accessed on 1 March 2025). All three sgRNAs were located in the first exon of *szlA/szlB*, which was selected to increase the probability of generating loss-of-function mutations at the early coding region of the gene. The sgRNA target sites were designated as *szl*-sgRNA1, *szl*-sgRNA2, and *szl*-sgRNA3, respectively (Figure 1). The sequences and genomic information of the sgRNAs are listed in Table S1.

The sgRNAs were generated as described previously with minor modifications [26]. Briefly, sgRNA templates were prepared by PCR amplification using target-specific oligonucleotides containing the T7 promoter sequence. The sgRNAs were then transcribed in vitro using the MEGAshortscript T7 Transcription Kit (Thermo Fisher Scientific, Waltham, MA, USA) according to the manufacturer’s instructions. The synthesized sgRNAs were purified by phenol–chloroform extraction, precipitated with ethanol, and dissolved in RNase-free water. The quality and concentration of purified sgRNAs were evaluated by agarose gel electrophoresis and spectrophotometric analysis before microinjection.

### 3.3. Microinjection of sgRNA/Cas9 Ribonucleoprotein Complexes

For CRISPR/Cas9-mediated mutagenesis, each *szl* sgRNA was mixed with Cas9 protein to prepare the injection solution. Briefly, Cas9 protein (300 ng/μL; New England Biolabs, Ipswich, MA, USA) and sgRNA (300 ng/μL) were diluted in injection buffer containing 0.5% phenol red, 20 mM HEPES, and 150 mM KCl. The sgRNA/Cas9 mixture was incubated at 37 °C for 15 min before microinjection to facilitate formation of ribonucleoprotein complexes. Approximately 5 nl of the prepared injection solution was delivered into the animal pole of one-cell-stage goldfish embryos using a NANOLITER 2020 microinjector (World Precision Instruments, Sarasota, FL, USA). Microinjection was performed within approximately 10–15 min after fertilization. After injection, embryos were transferred to filtered freshwater and maintained at 23 °C. At 12 h post-injection, unfertilized, dead, or morphologically abnormal embryos were removed manually. Hatched larvae were reared under standard conditions and fed with *Paramecium* at the early larval stage, followed by newly hatched brine shrimp during subsequent development.

### 3.4. Detection of Targeted Mutations by Sanger Sequencing

To evaluate the mutagenic efficiency of each *szl* sgRNA, injected embryos were sampled at 1 day post-fertilization (dpf). For each sgRNA group, 30 embryos were randomly selected for genomic DNA extraction. Genomic DNA was prepared using a Proteinase K-based lysis method. Briefly, embryos were lysed in 20 μL of DNA extraction buffer, heated at 100 °C for 5 min, and immediately chilled on ice for 2 min. Proteinase K was then added to a final amount of 1 μL per sample using a 10 mg/mL stock solution, followed by incubation at 55 °C for 2 h. The lysates were subsequently heated at 100 °C for 5 min to inactivate Proteinase K and used directly as PCR templates. Genomic fragments spanning each sgRNA target site were amplified using 3G Taq Master Mix for PAGE (Vazyme Biotech Nanjing, China) according to the manufacturer’s protocol. Target-specific primers flanking the edited regions were designed for each sgRNA site, and the primer sequences are provided in Table S1. PCR products with the expected size were purified and subjected to Sanger sequencing using an Applied Biosystems 3730xl DNA Analyzer (Applied Biosystems, Foster City, CA, USA). The resulting sequencing chromatograms were analyzed to identify CRISPR/Cas9-induced insertions and deletions around the predicted cleavage sites. Mutation profiles and editing efficiencies were estimated using the Inference of CRISPR Edits (ICE) analysis tool (version 4.0; EditCo Bio (Redwood City, CA, USA); https://store.editco.bio/tools/ice; accessed on 1 March 2025), which decomposes mixed Sanger sequencing traces to infer the position, type, and relative frequency of indel mutations generated at each target site. [27].

### 3.5. Phenotypic Observation and Skeletal Staining

To evaluate the morphological consequences of CRISPR/Cas9-mediated *szl* disruption, larvae were examined at the posterior swim bladder stage for caudal fin-fold morphology and at the anal fin ray stage for mineralized skeletal patterning. WT and edited specimens included in each comparison were stage-matched, and the viewing orientation was recorded during imaging. Representative larvae showing normal single-tail morphology or twin-tail-like caudal bifurcation were photographed using a Leica M205 FA fluorescence stereomicroscope (Leica Microsystems GmbH, Wetzlar, Germany). For visualization of mineralized skeletal structures, larvae were subjected to calcein staining according to previously reported fluorescence-based skeletal observation methods with minor modifications. Briefly, live larvae were immersed in freshly prepared 0.2% calcein solution diluted in filtered freshwater and incubated for 10 min in the dark. After staining, larvae were rinsed three times with filtered freshwater, 5 min each, to remove excess background fluorescence. The stained larvae were then transferred to clean freshwater and observed using a Leica M205 FA fluorescence stereomicroscope (Leica Microsystems GmbH, Wetzlar, Germany) with the fluorescein/GFP channel. The caudal region was carefully examined to assess mineralized caudal fin rays, caudal axial skeletal elements, vertebral structures, and fin-supporting skeletal components.

### 3.6. Quantitative Analysis of Dorsal–Ventral Axis-Related Gene Expression After szl Disruption

To investigate whether CRISPR/Cas9-mediated *szl* disruption affected the dorsal–ventral axis-related regulatory network, quantitative real-time PCR was performed to analyze the expression levels of *chdA*, *chdB*, *noggin1*, *noggin2*, *bmp2*, *bmp4*, *eve1*, and *gsc* in control and *szl*-edited fish. For each larva exhibiting a clear bifurcated caudal phenotype, the caudal tissue was dissected for total RNA extraction, whereas the remaining trunk tissue from the same individual was used for genomic DNA extraction and mutation analysis. The target regions of both *szlA* and *szlB* were amplified and analyzed by Sanger sequencing, and only larvae carrying detectable mutations at the corresponding target sites of both duplicated genes were included in the qPCR analysis. For each edited biological replicate, caudal tissues from three selected F0 larvae were pooled, and three to four independently prepared pooled samples were analyzed together with independently prepared control samples. Samples were collected at Asb stage, immediately frozen in liquid nitrogen, and stored at −80 °C until RNA extraction. Total RNA was extracted using an RNA extraction kit (Vazyme, China) according to the manufacturer’s instructions. RNA concentration and purity were assessed using a spectrophotometer, and RNA integrity was evaluated by agarose gel electrophoresis. First-strand cDNA was synthesized from total RNA using the HiScript III 1st Strand cDNA Synthesis Kit (Vazyme, China) following the manufacturer’s protocol. Quantitative real-time PCR was performed using ChamQ SYBR Color qPCR Master Mix (Vazyme, China) on an ABI 7500 Real-Time PCR System (Thermo Fisher Scientific, USA). Gene-specific primers were used to amplify *chdA*, *chdB*, *szlA*, *szlB*, *noggin1*, *noggin2*, *bmp2*, *bmp2*, *eve1*, and *gsc*, and the primer sequences are listed in Table S1. The *β-actin* gene was used as the internal reference for normalization. The specificity of each amplification reaction was verified by melting-curve analysis. Relative expression levels were calculated using the 2^−ΔΔCt^ method. Statistical analyses were performed using Prism 10. Data are presented as mean ± standard error of the mean unless otherwise indicated. Differences between groups were considered statistically significant at *p* < 0.05.

## 4. Conclusions

In conclusion, CRISPR/Cas9-mediated disruption of conserved coding regions shared by the duplicated *szlA* and *szlB* genes was sufficient to induce twin-tail-like caudal bifurcation in single-tail goldfish larvae. The effective sgRNAs generated high-frequency indels, early fin-fold bifurcation, persistent separation of caudal fin rays, and selective alterations in Chordin/BMP-related gene expression. These findings provide genomic-level evidence that *szl* contributes to median caudal patterning and represents an alternative developmental route to caudal bifurcation beyond the classical *chdA* mutation.

## Data Availability

The data presented in this study are available on request from the corresponding authors.

## Supplementary Materials

The following supporting information can be downloaded at: https://www.mdpi.com/article/10.3390/ijms27167318/s1.
