# Supplementary material for: CRISPR/Cas9-Mediated Disruption of Duplicated Sizzled Genes Induces Twin-Tail-like Caudal Bifurcation in Goldfish (Carassius auratus)
Source: Int J Mol Sci. 2026 Aug 16;27(16):7318. doi: 10.3390/ijms27167318 (PMC13513690; doi:10.3390/ijms27167318)
Supplement: Supplementary file 1 [file ijms-27-07318-s001.zip › ijms-4452802-Figure S1.pdf]

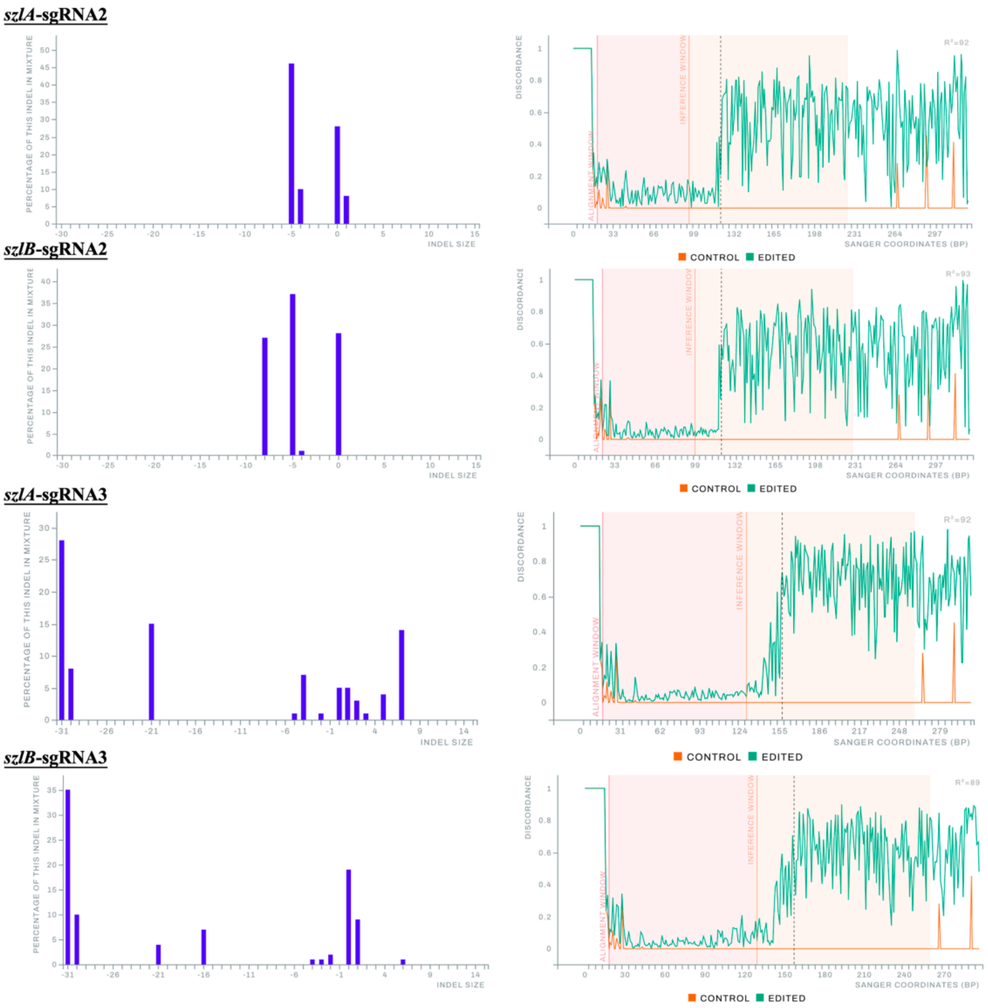

Figure S1. Indel size distribution and ICE discordance profiles of CRISPR/Cas9-induced mutations at the duplicated goldfish *szlA* and *szlB* loci. ICE analysis showing the indel size distribution and sequence discordance profiles generated by *szl*-sgRNA2 and *szl*-sgRNA3 at the endogenous *szlA* and *szlB* target loci. The left panels show the relative proportion of each inferred indel class within the edited allele mixture. The right panels show ICE discordance plots comparing control and edited Sanger traces across the target regions. Green lines represent edited samples, orange lines represent control samples, and shaded regions indicate the ICE inference windows used for indel decomposition. The sharp increase in discordance downstream of the predicted Cas9 cleavage sites confirms efficient CRISPR/Cas9-mediated mutagenesis at both duplicated *szl* paralogues. The high ICE model fitting values further support the reliability of the inferred indel profiles.
