# Supplementary material for: CRISPR/Cas9-Mediated Disruption of Duplicated Sizzled Genes Induces Twin-Tail-like Caudal Bifurcation in Goldfish (Carassius auratus)
Source: Int J Mol Sci. 2026 Aug 16;27(16):7318. doi: 10.3390/ijms27167318 (PMC13513690; doi:10.3390/ijms27167318)
Supplement: Supplementary file 1 [file ijms-27-07318-s001.zip › ijms-4452802-Table S1.pdf]

**Table S1. Specific primers used in this study**		
**Primer name**	**Sequence (5'-3')**	**Usage**
szl-sgRNA1-F	GATCACTAATACGACTCACTATAGGGTTCCTCGCTCGGACGACGTTTTAGAGCTAGAAAT	sgRNA synthesis
szl-sgRNA2-F	GATCACTAATACGACTCACTATAGGGCTCATCTGCGGCGGGACGTTTTAGAGCTAGAAAT	sgRNA synthesis
szl-sgRNA3-F	GATCACTAATACGACTCACTATAGGGCAGACGCATCTCTGAGTAGTTTTAGAGCTAGAAAT	sgRNA synthesis
szl-mut-F1	TTATCTCCCGCTCGTCACTGTCTG	verify primer for *sgRNA1/2/3*
szl-mut-R1	ACACACACCTGAGCTCAGAGAGAG	verify primer for *sgRNA1/2/3*
RT-chdA-F	CGGAGAGAAGGAAAATGCTG	specific primers for RT-qPCR
RT-*chdA*-R	GTGGGATTATGAACGCTGAG	specific primers for RT-qPCR
RT-*chdB*-F	TTACCCATCCATCCTGAGAC	specific primers for RT-qPCR
RT-*chdB*-R	AATGCATCACACCGAACG	specific primers for RT-qPCR
RT-*nog1*-F	AGTATCCCATCATCACCGAG	specific primers for RT-qPCR
RT-*nog1*-R	CTCATGTCCAGAAATACGACAG	specific primers for RT-qPCR
RT-*nog2*-F	GAGTATTTCTGACACAGCGG	specific primers for RT-qPCR
RT-*nog2*-R	GCGTCTAGAACATGTTCGG	specific primers for RT-qPCR
RT-*bmp2*-F	GACTCTCTGTCGTGGGATAA	specific primers for RT-qPCR
RT-*bmp2*-R	GACCATGTTCAGTCAGTTCC	specific primers for RT-qPCR
RT-*bmp4*-F	CTCTCGACAAACATGGACTG	specific primers for RT-qPCR
RT-*bmp4*-R	AGACTAGCATAGCTGCTCTC	specific primers for RT-qPCR
RT-*evel*-F	TCAGGACTATGAGGAGCTTG	specific primers for RT-qPCR
RT-*evel*-R	GATCACTGTCAGCATGAGC	specific primers for RT-qPCR

RT-*gsc*-F	AAAGTAACGTGGATTCTGGC	specific primers for RT-qPCR
RT-*gsc*-R	CATGTCTTCAGTTACAGCCC	specific primers for RT-qPCR
β-actin-F	GTGATGGATTCTGGTGATGGTGTG	internal primers for RT-qPCR
β-actin-R	TCATCAGGTAGTCTGTCAGGTCACG	internal primers for RT-qPCR
